# Patterns of venous collateral development after splenic vein occlusion associated with surgical and oncological outcomes after distal pancreatectomy

**DOI:** 10.1002/ags3.12830

**Published:** 2024-06-17

**Authors:** Keishi Sugimachi, Tomonari Shimagaki, Takahiro Tomino, Emi Onishi, Yohei Mano, Tomohiro Iguchi, Masahiko Sugiyama, Yasue Kimura, Masaru Morita, Yasushi Toh

**Affiliations:** ^1^ Department of Hepatobiliary and Pancreatic Surgery NHO Kyushu Cancer Center Fukuoka Japan; ^2^ Department of Gastroenterological Surgery NHO Kyushu Cancer Center Fukuoka Japan

**Keywords:** distal pancreatectomy, left‐sided portal hypertension, pancreatic ductal adenocarcinoma, splenic vein occlusion

## Abstract

**Aims:**

Splenic vein occlusion (SpVO) due to a pancreatic tumor may result in the development of collateral circulation and left‐sided portal hypertension. This study aimed to investigate the impact of SpVO on distal pancreatectomy (DP) and provide insights about the management of such cases.

**Methods:**

This retrospective analysis included 124 patients who underwent DP from 2014 to 2022. A subgroup analysis was performed on 88 patients who underwent DP for pancreatic ductal adenocarcinoma (PDAC).

**Results:**

SpVO was found in 26 (20.8%) patients. The patients with SpVO had significantly larger splenic volumes and lower platelet counts. Compared to the patients with patent splenic veins (SpVs), the patients with SpVO underwent significantly longer operations (*p* = 0.006), with a higher incidence of postoperative complications (*p* = 0.002). We classified the collateral routes associated with SpVO into five patterns. The most common pattern was the left gastroepiploic vein type, which was associated with a tumor of the pancreatic body. In patients with PDAC, SpVO was associated with larger tumors, microscopic vascular permeation, and peritoneal recurrence. However, the differences between overall and recurrence‐free survival rates in the patients with SpVO vs those with patent SpVs were not significant.

**Conclusions:**

SpVO causes left‐sided portal hypertension, which can be a risk for perioperative complications in DP. Operative planning based on the classification of collateral flow patterns may help prevent intraoperative congestion and perioperative complications.

## Graphical abstract

This study showed that splenic vein occlusion causes left‐sided portal hypertension, which can be a risk for perioperative complications in distal pancreatectomy. Our findings could contribute to the operative planning based on the classification of collateral flow patterns and may help prevent intraoperative congestion and perioperative complications.

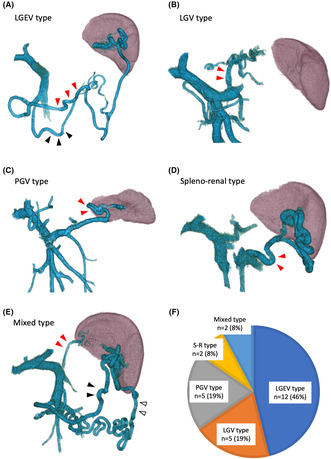

## 1 INTRODUCTION

Distal pancreatectomy (DP), formally “left radical pancreatectomy,” is an established surgical procedure that involves removal of the distal (left) part of the pancreas, with or without splenectomy. DP is typically performed to remove tumors located in the left side or tail of the pancreas, including benign tumors, pancreatic ductal adenocarcinoma (PDAC), and cystic tumors. DP has traditionally been performed via an open approach. Since minimally invasive DP was first performed in 1994,^1^ DPs via laparoscopic and robot‐assisted approaches have rapidly gained in popularity and are increasingly used.^2^

The splenic vein (SpV) originates from the spleen, passes through the left side of the pancreas, and primarily drains into the portal venous system. Pancreatic tumors and pancreatitis that lead to DP often cause SpV occlusion (SpVO)^3^; therefore, it is essential to be mindful of the presence of SpVO when performing a DP. SpVO is associated with several critical conditions that can be encountered during DP, including the presence of collateral vessels and left‐sided portal hypertension (LSPH).^4^ The development of collateral circulation and congestion around the pancreas and spleen confers an increased risk of marked intraoperative bleeding due to vascular injury.

LSPH primarily affects the spleen and its surrounding vessels and is usually asymptomatic. Liver function also remains normal.^5^ LSPH leads to splenomegaly due to increased blood flow and congestion, and the formation of collateral vessels sometimes leads to the formation of varicose veins in the stomach (gastric varices).^4^, ^6^, ^7^ Therefore, the assessment and understanding of the anatomy of the spleen and pancreas and associated collateral circulation is crucial for avoiding perioperative complications.

There are several challenges and questions that need to be addressed regarding DPs for patients with SpVO. First, it is essential to determine whether the presence of SpVO affects perioperative outcomes such as intraoperative bleeding and postoperative complications. If SpVO poses perioperative risks, a safe DP procedure requires deciding on which preoperative preparations, intraoperative surgical procedures, and postoperative approaches for management are essential. Understanding how to mitigate the risks associated with SpVO is paramount for the overall success and safety of the procedure. Second, an SpVO must be assessed in relationship to its effect on the pathophysiology and prognosis of a patient's PDAC. PDAC is one of the most lethal cancers worldwide because it is difficult to diagnose during its early stages and because of its highly malignant potential.^8^ Surgical resection is the only curative option for patients with PDAC; however, the prognosis after resection remains poor.^9^

If SpVO associated with PDAC impacts disease progression and outcomes, it becomes imperative to clarify the nature of these effects in order to develop comprehensive treatment plans and estimate prognosis. Addressing these challenges should contribute to improved patient care and surgical decision‐making for performing DPs in patients with PDAC associated with SpVO.

The effect of SpVO on DP has only been sporadically reported via imaging analysis, case reports, and a limited number of case studies.^3^, ^6^, ^7^, ^10^, ^11^, ^12^ However, to our best knowledge, there has not been a systematic and consolidated study on a substantial number of cases.

Given the importance of assessing risk factors for DP in patients with SpVO, we conducted a retrospective study of patients who underwent DP to investigate the impact of the clinicopathological parameters associated with SpVO on those patients. We thought that these parameters could be useful for establishing safe approaches for DP. We also aimed to elucidate the oncological impacts of SpVO on patients with PDAC that might aid the development of treatment strategies for improving outcomes.

## 2 METHODS

### 2.1 Patients

This was a retrospective single‐center study of 133 patients who underwent DP from January 2014 to June 2023 at Kyushu Cancer Center. Prospectively collected and maintained data were analyzed. After the exclusion of nine patients who underwent spleen‐preserving DP, the remaining 124 cases were included in the analysis. The median age of the patients was 70 (24─85) y. The study patients had the following conditions: PDAC, *n* = 88 (71%) patients; intraductal papillary neoplasms, *n* = 11 (9%); neuroendocrine tumors, *n* = 11 (9%), and other diseases *n* = 14 (11%). This study was approved by the Institutional Review Board of Kyushu Cancer Center (2019‐54).

### 2.2 Surgical procedures and assessment

The surgical approaches included 81 open surgeries and 43 minimally invasive surgeries that comprised 29 laparoscopic surgeries and 14 robot‐assisted surgeries. Among the 124 patients, a combined portal vein (PV) and superior mesenteric vein (SMV) resection was performed in two (1.6%), and a combined portal vein and other‐organ (stomach, colon, kidney) resection was performed in 10 (8%). Transection of parenchymal pancreas was performed by a surgical stapler. Conventional pancreatectomy was performed for patients with benign tumors or PDAC without peripancreatic infiltration; radical antegrade modular pancreatosplenectomy was performed for patients with PDAC associated with peripancreatic infiltration.^13^ Systematic lymph node dissection was performed for the patients with PDAC according to the General Rules for the Study of Pancreas Cancer (7th edition), as edited by the Japan Pancreas Society.^14^ Postoperative pancreatic fistula (POPF) and delayed gastric emptying (DGE) were identified in accordance with the standards used by the International Study Group of Pancreatic Surgery,^15^, ^16^ and the Clavien–Dindo classification was used for grading complications.^17^

### 2.3 Perioperative treatment

Upfront surgery was performed prior to 2019, and neoadjuvant chemotherapy (NAC) with gemcitabine and S‐1 was applied since 2019. As for adjuvant chemotherapy, treatment with S‐1 was the standard protocol for all cases if the patient's general condition permitted. However, the decision on whether to proceed with treatment also depended on patient consent and the attending physician's decision in this retrospective analysis. There were no patients who underwent chemoradiotherapy.

### 2.4 Imaging analysis of SpVO, collateral veins, and splenic volumetry

All patients underwent 1‐to‐2‐mm slice‐thickness abdominal contrast‐enhanced multidetector‐row computed tomography (CT) within 2 mo before surgery. SpVO and collateral veins emanating from the spleen were traced and defined on serial transaxial or reconstructed 3‐D images. These 3‐D images were created by specialized radiologists and dedicated medical radiological technicians. The image evaluation of collateral circulation was confirmed by multiple pancreatic surgeons (K.S., T.S., T.T., and E.O.). The collateral pathway was classified based on the evident main route even if there was a narrow, minor secondary vessel. Cases with multiple collateral routes where it was not possible to determine which one was the main pathway were classified as mixed type. Volume measurement of the spleen was performed by drawing the outline of the spleen manually in three dimensions with the use of a 3‐D image analysis system (Synapse Vincent, Fujifilm, Tokyo, Japan).

### 2.5 Subgroup analysis of PDAC cases

We performed subgroup analysis of 88 patients with PDAC. In this study we used General Rules for the Study of Pancreas Cancer (7th edition) edited by the Japan Pancreas Society,^14^ and the TNM classification system of malignant tumors by the International Union Against Cancer (8th edition)^18^ for the histopathological evaluation of surgical specimens. R0 curative resection was defined as the absence of tumor cells in the resection margins of the resected or dissected specimen. Postoperative follow‐up was conducted every 3 mo by routine blood testing and imaging by either contrast‐enhanced CT or magnetic resonance imaging. The median follow‐up period of SpVO and patent SpV groups were 25.1 [2–97] and 30.0 [1–111] mo, respectively.

### 2.6 Statistical analysis

Associations between categorical variables were determined by the *χ* ^2^ or Fisher's exact tests. Continuous data are presented as means ± standard deviation or medians (range). Comparisons between continuous variables were analyzed by Student's *t* test for normally distributed data, and the Mann–Whitney *U* test for non‐normally distributed data. Overall survival (OS) and disease‐free survival (DFS) were estimated by the Kaplan–Meier method and compared by the log‐rank test. A *p*‐value of <0.05 was considered statistically significant. JMP Pro 16 software (SAS Institute, Cary, NC, USA) was used for statistical analysis.

## 3 RESULTS

### 3.1 SpVO due to pancreatic tumor

SpVO was found in 26 (20.8%) of 124 patients. Table 1 shows the clinicopathological factors of patients with patent SpVs compared with those of patients with SpVO. The hemoglobin levels and platelet counts were significantly lower in patients with SpVO compared to patients with patent SpVs. The splenic volumes as measured by 3‐D‐CT volumetry analysis were significantly larger in patients with SpVO. These data indicated that LSPH was caused by SpVO. The percentages of patients in each group undergoing minimally invasive surgery (laparoscopic or robot‐assisted surgery) were similar, but the rate of multivisceral resection of organs other than the pancreas and spleen was significantly higher in the patients with SpVO.

**TABLE 1:** Comparison of clinicopathological parameters in patients with/without splenic vein occlusion.

Factors	Patent SpV (*n* = 98)	SpV occlusion (*n* = 26)	*p* Value
Age (y)	68.2 ± 1.2	66.3 ± 2.3	0.472
Sex (male/female)	58/40	10/16	0.059
Tumor type			
PDAC	65 (66%)	23 (88%)	0.018
Others	33 (34%)	3 (12%)	
Tumor–main location			
Body	56 (57%)	7 (27%)	0.005
Tail	42 (43%)	19 (73%)	
Tumor size (cm)	2.4 ± 0.2	3. 8 ± 0.3	<0.001
White blood cell count (×103/μL)	5.25 ± 0.2	5.50 ± 0.47	0.573
Hemoglobin (g/dL)	12.9 ± 0.1	12.4 ± 0.3	0.029
Platelet (×10^4^/μL)	20.7 ± 0.5	18.4 ± 1.1	0.027
CEA (ng/mL)	3.1 ± 0.3	3.3 ± 0.6	0.384
CA19‐9 (U/mL)	116 ± 37	247 ± 70	0.051
Splenic volume (mL)	123 ± 6.5	157 ± 13	0.009
Preoperative chemotherapy			
Yes	19 (19%)	17 (65%)	<0.001
Surgery type			
Open	65 (66%)	16 (62%)	0.650
MIS	33 (34%)	10 (38%)	
Multivisceral resection			
Yes	4 (4%)	6 (23%)	0.005

Abbreviations: CA19‐9, carbohydrate antigen 19–9; CEA, carcinoembryonic antigen; MIS, minimally invasive surgery; PDAC, pancreatic ductal adenocarcinoma; SpV, splenic vein.

### 3.2 Postoperative outcome of SpVO cases

Table 2 shows comparisons of the operative parameters for patients with/without SpVO. Both operative times and blood loss tended to be higher in patients with SpVO, although the differences were not significant. These results indicated that patients with SpVO underwent more complicated surgical procedures than patients with patent SpVs. The patients with SpVO had a significantly higher rate of postoperative Clavien–Dindo grade 3 or more complications, although the rates of POPF were comparable. The grade 3 complications in patients with SpVO were as follows: intraabdominal infection in three patients, POPF in two, and pneumothorax in one. There were no grade 4 and 5 complications. The platelet counts were significantly higher in patients with than without SpVO on postoperative d 7, which is a reversal of the platelet counts before surgery. The platelet counts were similar in the two patient groups on postoperative day 90.

**TABLE 2:** Operative parameters in patients with/without splenic vein occlusion.

Factors	Patent SpV (*n* = 98)	SpV occlusion (*n* = 26)	*p* Value
Operative time (min)	250 ± 8.2	279 ± 16	0.056
Blood loss (g)	170 ± 26	259 ± 51	0.062
Clavien–Dindo complications Grade ≥3	3 (3%)	6 (23%)	0.002
POPF Grade B or C	13 (13%)	5 (19%)	0.456
Postoperative hemorrhage	1 (1%)	0 (0%)	0.490
DGE Grade B or C	4 (4%)	1 (4%)	0.978
Mortality	0 (0%)	0 (0%)	―
Platelet 7d (×10^4^/μL)	34.2 ± 1.0	37.4 ± 1.9	0.011
Platelet 90d (×10^4^/μL)	26.1 ± 1.1	26.1 ± 2.1	0.492
Postoperative adjuvant chemotherapy	46 (47%)	20 (77%)	0.006

Abbreviations: d, day; DGE, delayed gastric emptying; POPF, postoperative pancreatic fistula; SpV, splenic vein.

### 3.3 Collateral venous flow patterns and operative outcomes in patients with SpVO

**FIGURE 1:**
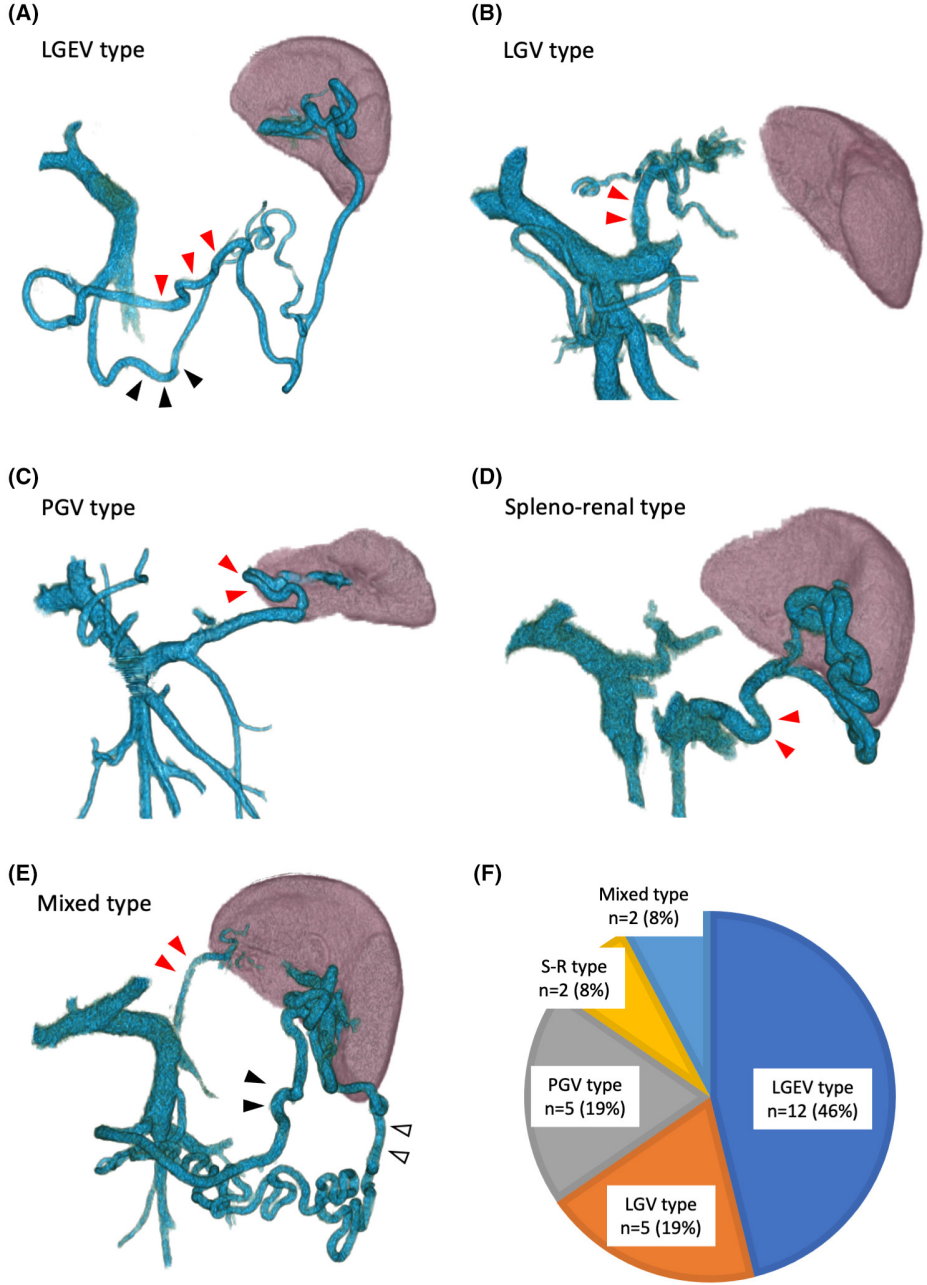
Classification of five patterns of collateral circulation due to splenic vein occlusion. (A) Left gastroepiploic vein (LGEV) type. The LGEV (red arrowheads) and/or colic vein (black arrowheads) comprise the main collateral route, and collateral flow exists at the greater curvature of the stomach and drains into the superior mesenteric vein. (B) Left gastric vein (LGV) type. The LGV (red arrowheads) is the main collateral flow and drains into the portal vein (PV) or proximal splenic vein. (C) Posterior gastric vein (PGV) type. PGV or short gastric vein (red arrowheads), which drain into the splenic vein, comprise the main collateral route. (D) Splenorenal type. The splenorenal shunt (red arrowheads) is the main collateral route. (E) Mixed type. Patients with more than one of the described collateral routes that exist concurrently. LGV (red arrowheads), LGEV (black arrowheads), and spleno‐systemic shunt (white arrowheads) comprise the collateral routes. (F) Pie chart showing the frequency of the five patterns in 26 patients with splenic vein occlusion.

We analyzed the splenic venous flow patterns of 26 patients with SpVO in a preoperative imaging study. Collateral flow in two (8%) patients drained into the systemic circulation (splenorenal shunt), and collateral flow in 24 (92%) patients drained into the portal venous system. We categorized the collateral routes into five patterns depending on the alternative main outflow from the spleen (Figure 1), as follows: (a) left gastroepiploic vein (LGEV) type (12 [46%] patients, Figure 1A). LGEV and/or middle colic vein via the arc of Barkow are the main collateral routes. In these patients, collateral flow exists at the greater curvature of the stomach and drains into the SMV; (b) left gastric vein (LGV) type (five [19%] patients, Figure 1B). The LGV is the main collateral flow. In these patients, collateral flow drains into the PV or proximal SpV; (c) posterior gastric vein (PGV) type (five [19%] patients, Figure 1C). The PGV or short gastric vein, which drain into the SpV, make up the main collateral route. (d) Spleno‐renal type (two [8%] patients, Figure 1D). The splenorenal shunt is the main collateral route. (e) Mixed type (two [8%] patients, Figure 1E); these patients could not be classified. The frequency of the five patterns in 26 SpVO patients is shown in Figure 1F. We found that a longer operative time and more blood loss in LGEV type and PGV type compared to other cases (Table 3). Although the number of cases for each was limited, these results suggested that the presence of main collateral circulation around the spleen may increase the difficulty of surgery.

**TABLE 3:** Operative outcomes depending on collateral patterns.

Factors	LGEV type (*n* = 12)	LGV type (*n* = 5)	PGV type (*n* = 5)	S‐R type (*n*‐2)	Mixed type (*n* = 2)	*p* Value
Operative time (min)	281 [181–349]*	234 [170–333]^†^	337 [306–415]*, ^†^	261 [202–321]	260 [209–311]	0.016*, 0.007
Blood loss (g)	200 [60–930]	90 [20–108]	210 [90–2000]	25 [20–30]	112 [25–200]	ns
Clavien–Dindo complications Grade ≥3	3 (25%)	2 (40%)	1 (20%)	0 (0%)	0 (0%)	ns
POPF Grade B or C	2 (17%)	2 (40%)	1 (20%)	0 (0%)	0 (0%)	ns

Abbreviations: LGEV, left gastroepiploic vein; LGV, left gastric vein; ns, not significant; PGV, posterior gastric vein; POPF, postoperative pancreatic fistula; S‐R, splenorenal shunt.

* LGEV type versus PGV type.

† LGV type versus PGV type.

### 3.4 Association between collateral venous flow patterns and tumor locations

**FIGURE 2:**
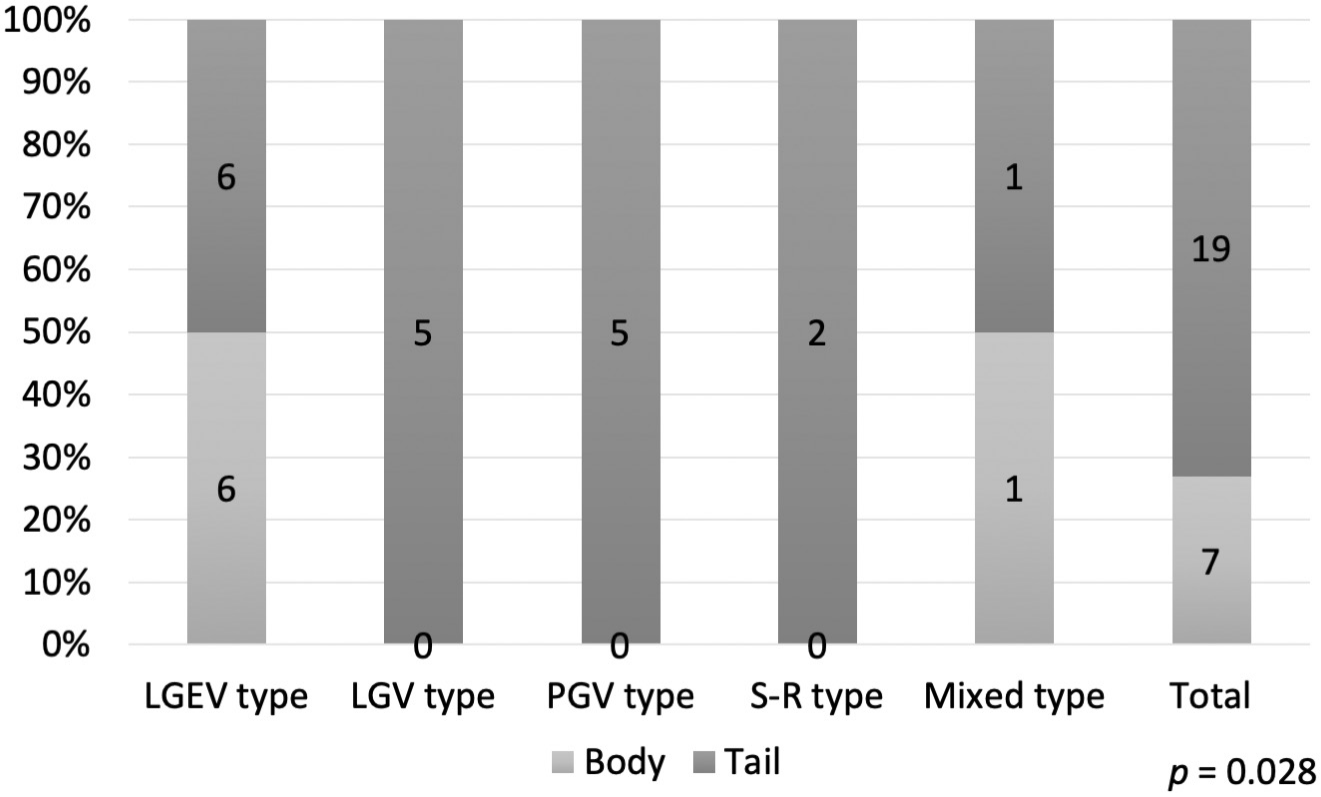
Correlation between collateral patterns and main location of pancreatic tumor in 26 patients with splenic vein occlusion. LGEV, left gastroepiploic vein; LGV, left gastric vein; PGV, posterior gastric vein; S‐R, splenorenal shunt.

We then analyzed the associations between collateral patterns and the main locations of the tumors. In tumors of the pancreatic body, collateral circulations that flowed into the SMV through the LGEV and colic veins in the greater curvature of the stomach were significantly more common (Figure 2, *p* = 0.028). Conversely, LGV‐type, PGV‐type, and splenorenal type tumors were all tumors of the pancreatic tail (Figure 2). We analyzed the anatomy of the LGV in the 26 SpVO cases. LGV flows into PV in 11 cases, and SpV in 15 cases (with four cases being occluded). Focusing on the pancreatic body tumors in seven cases, LGV flows into PV in two cases, and SpV in five cases (with four cases being occluded). All three cases of body tumors with patent LGV were categorized as the LGEV pattern. These results suggested that LGEV was the first collateral route, regardless of the patency of the LGV.

### 3.5 Oncological outcomes of patients with SpVO and PDAC

**FIGURE 3:**
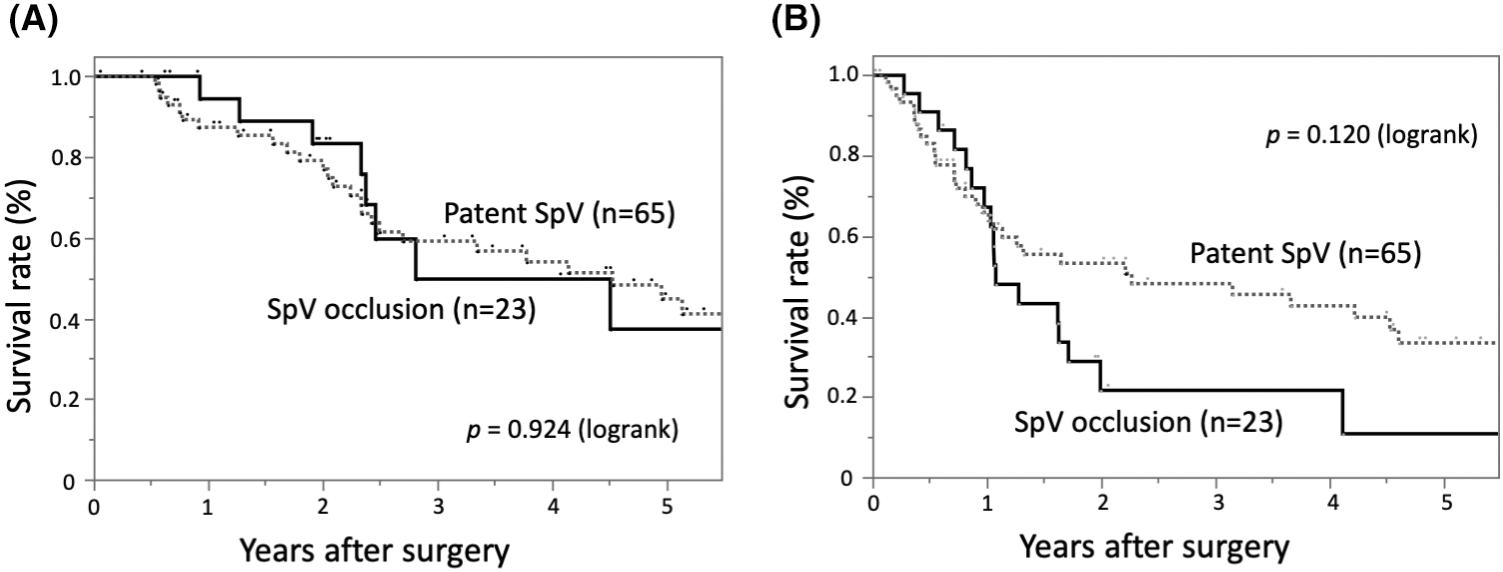
Kaplan–Meier curves of (A) overall survival and (B) disease‐free survival of patients who underwent distal pancreatectomy for pancreatic ductal adenocarcinoma stratified by splenic vein occlusion or patent splenic veins. SpV, splenic vein.

The oncological clinicopathological factors of 23 PDAC patients with SpVO and 65 PDAC patients with patent SpVs were compared (Table 4). Neoadjuvant and adjuvant chemotherapy were more frequently administered to patients with SpVO. The diameters of tumors were significantly larger, and microscopic vascular permeation was more frequently found in patients with SpVO. During the follow‐up period, 32 of 65 (49%) patients with patent SpVs, and 17 of 23 (74%) patients with SpVO (74%) had recurrent disease. Liver metastases were more frequently found in patients with patent SpVs, and peritoneal metastases were more common in patients with SpVO. We compared the OS and DFS rates of the patients with SpVO and those of the patients with patent SpVs. Both groups obtained comparable rates of OS (Figure 3A) and DFS (Figure 3B). Although the difference in background factors is a limitation of this retrospective study, it still suggests that SpVO alone may not be an unresectable factor, but when combined with chemotherapy, it may indicate a favorable prognosis comparable to the patent SpV cases.

**TABLE 4:** Comparison of oncological parameters in PDAC patients with/without splenic vein occlusion.

Factors	Patent SpV (*n* = 65)	SpV occlusion (*n* = 23)	*p* Value
Neoadjuvant chemotherapy			
Yes	16 (25%)	16 (70%)	<0.001
Adjuvant chemotherapy			
Yes	45 (69%)	20 (87%)	0.030
Tumor diameter (cm)	2.2 [0.4–8.4]	3.2 [1.1–6.7]	<0.001
Histological differentiation			
cis	6 (9%)	0 (0%)	0.248
Well	3 (5%)	1 (4%)	
Mod	24 (37%)	11 (48%)	
Poor	32 (49%)	11 (48%)	
Pathological T factor			
0	4 (6%)	1 (4%)	0.362
1	13 (20%)	1 (4%)	
2	2 (3%)	1 (4%)	
3	43 (66%)	18 (78%)	
4	3 (5%)	2 (9%)	
Lymph node metastases			
Yes	31 (48%)	15 (65%)	0.145
Microscopic lymphatic invasion			
Yes	6 (9%)	3 (13%)	0.612
Microscopic vascular invasion			
Yes	18 (28%)	14 (61%)	0.005
Microscopic neural invasion			
Yes	48 (74%)	19 (83%)	0.386
Pathological R0 resection			
Yes	62 (95%)	20 (87%)	0.195
Recurrence			
Yes	32 (49%)	17 (74%)	0.037
Recurrence site			
Local	6 (19%)	0 (0%)	0.036
Remnant pancreas	4 (13%)	4 (24%)	
Peritoneum	3 (9%)	5 (29%)	
Liver	14 (44%)	4 (24%)	
Lung	5 (16%)	3 (18%)	
Lymph nodes	0 (0%)	1 (6%)	

Abbreviations: PDAC, pancreatic ductal adenocarcinoma; SpV, splenic vein; cis, carcinoma in situ.

## 4 DISCUSSION

To our best knowledge, this study is the first to show the collateral patterns and clinical outcomes of patients with SpVO who underwent DP. When the SpV is occluded, collateral pathways are formed to compensate for impaired drainage of the splenic vein. We herein identified the five following major pathways: the left gastroepiploic veins, which run along the greater curvature of the stomach, and colonic vein (LGEV type); left gastric vein (LGV type); posterior or short gastric veins (PGV type); and splenorenal shunt (S‐R type). We also found that SpVO due to pancreatic tumor can lead to increased pressure in the veins surrounding the spleen and pancreas, resulting in LSPH, which is clinically manifested as splenomegaly and hypersplenism (thrombocytopenia). Over the long term, LSPH can lead to clinically problematic conditions such as gastric varices. However, all the patients in this study underwent DP; thus, patients with LSPH caused by SpVO were radically treated by splenectomy. In fact, the postoperative platelet counts actually remained at similar levels regardless of the preoperative presence of SpVO, indicating that there were no long‐term postoperative clinical manifestations. The two main issues we aimed to elucidate in this study of PDAC patients with/without SpVO were as follows: first, to clarify the collateral circulation patterns that allow safe performance of DP in patients with SpVO; and second, to determine whether SpVO due to PDAC is a clinically concerning condition from an oncological perspective. By systematically examining the detailed patterns of collateral circulation in this study, we aimed to identify a safe and effective approach for performing DP in patients with SpVO. Enhancement of surgical safety means prioritizing the blockage of inflow, which is the splenic arterial blood flow, before dissecting the collateral vessels to prevent congestion of the organ and intraoperative bleeding.^19^ The preoperative recognition of the collateral pattern and a preoperative surgical plan are essential for efficiently performing the preemptive occlusion of inflow.

We thus propose methods for safely performing DP, which are based on different patterns of collateral flow due to SpVO. LGEV type: minimize dissection of the gastrocolic ligament and occlude blood flow of the splenic artery (SpA) before performing manipulations around the spleen. LGV type: pay attention to patients whose LGV exists anterior to the SpA, making early inflow occlusion difficult. Consider alternative procedures such as occlusion of the SpA via a retroperitoneal approach or clamping the SpA at the suprapancreatic portion of the pancreatic tail.^20^, ^21^ PGV type: avoid dissection of the gastrosplenic ligament and performing manipulations around the spleen, until the SpA is clamped. Splenorenal type: avoid a retroperitoneal‐first approach. In cases of buried SpA in the pancreas parenchyma,^22^, ^23^ it is necessary to consider preforming occlusion of the SpA via a retroperitoneal approach or clamping the SpA at the suprapancreatic portion of the pancreatic tail. The recommendations are intended to optimize the safety and efficacy of DP in patients with SpVO by strategically addressing the occlusion of arterial inflow based on the identified collateral circulation patterns.

SpVO is caused by compression or tumor invasion, leading to a higher prevalence of large tumors with extrapancreatic extension. We have observed that SpVO is more frequently associated with PDAC than other tumors, characterized by an increased incidence of extrapancreatic invasion. Analysis of the data specifically related to PDAC and comparison between patients with/without SpVO found that a larger tumor size and increased rate of microscopic vascular infiltration were observed in patients with SpVO. These data suggest that SpVO‐associated PDAC may be a more aggressive phenotype.

Recent reports have highlighted the utility of neoadjuvant chemotherapy in conjunction with surgery for borderline locally advanced resectable PDAC.^24^, ^25^ However, whether the same treatment strategy is applicable to SpVO‐associated PDAC has remained unclear. In this study, the differences between the postoperative OS and DFS rates of patients with/without SpVO were not significant; and patients with SpVO received significantly more preoperative and postoperative chemotherapy than the patients with patent SpVs. This result may reflect a higher prevalence of advanced tumors in the SpVO patients. Despite the SpVO cases having more advanced diseases, no statistically significant differences were observed in OS. One possible reason for this is the potential disparity in background factors between the SpVO and patent SpV groups in this retrospective analysis. Specifically, the rates of NAC and adjuvant chemotherapy were significantly higher in the SpVO group. In the Kaplan–Meier curve, there was a tendency towards lower DFS rate in the SpVO group, reflecting its higher recurrence rate; however, the difference was not significant. The relatively small sample size of the SpVO group and relatively short observation period may have influenced the results.

The data we presented in this study indicate that the combination of perioperative chemotherapy with curative resection is a standard treatment for PDAC patients with SpVO. However, the patterns of recurrence show that patients with SpVO have a higher incidence of peritoneal dissemination than patients with patent SpVs, indicating the need for careful intraoperative surgery, especially for the resection of large tumors.

The shortcomings of this retrospective study include limitations in establishing conclusive evidence for treatment strategies for SpVO‐associated PDAC, because of selection bias. Determining the optimal therapeutic approach for SpVO‐associated PDAC requires further prospective studies to validate our results.

In conclusion, to our best knowledge, this study provides new and important information on the significance of SpVO in patients who have undergone DP. Rather than assessing the presence or absence of SpVO, we thought that it was important to classify the anatomy and recognize the patterns of collateral circulation as major goals for preoperative planning that might be useful for safely performing DPs.

## AUTHOR CONTRIBUTIONS

K. Sugimachi participated in the conception and design of the study, and writing the article. T. Shimagaki, T. Tomino, E. Onishi, Y. Mano, and T. Iguchi participated in the acquisition of data. M. Sugiyama, Y. Kimura, and M. Morita participated in the statistical analysis and interpretation of data. M. Morita and Y. Toh participated in reviewing the article.

## FUNDING INFORMATION

This study was partly supported by JSPS KAKENHI (21 K08745) and the Uehara Memorial Foundation.

## CONFLICT OF INTEREST STATEMENT

Kimura Y, who is a coauthor of this article, is an editorial board member (upper digestive tract) of the *Annals of Gastroenterological Surgery*. The authors declare no conflicts of interest for this article.

## ETHICS STATEMENT

Approval of the research protocol: This study protocol complied with the ethical guidelines of human clinical research established by the Japanese Ministry of Health, Labour and Welfare as well as with the 1964 Helsinki Declaration and its later amendments. The study was approved by the Ethics and Indications Committee of the National Hospital Organization Kyushu Cancer Center (No. 2019‐54).

Informed Consent: Informed consent was obtained from each participant included in the study.

Registry and the Registration No. of the study/trial: N/A.

Animal Studies: N/A.
